# Injectable Pectin–Alginate Hydrogels for Improving Vascularization and Adipogenesis of Human Fat Graft

**DOI:** 10.3390/jfb14080409

**Published:** 2023-08-02

**Authors:** Ramu Janarthanan, Rangasamy Jayakumar, Subramania Iyer

**Affiliations:** 1 Department of Plastic and Reconstructive Surgery, Amrita Institute of Medical Sciences and Research Centre, Amrita Vishwa Vidyapeetham, Kochi 682041, India; janarthananr@aims.amrita.edu; 2 Polymeric Biomaterials Lab, School of Nanosciences and Molecular Medicine, Amrita Vishwa Vidyapeetham, Kochi 682041, India; rjayakumar@aims.amrita.edu

**Keywords:** fat grafting, hydrogel, injectability, alginate, pectin, adipogenesis, angiogenesis, PRP

## Abstract

Autologous fat grafting (AFG) is the most prevailing tool for soft tissue regeneration in clinics, although efficiency is limited to unpredictable volume resorption due to poor vascularization and eventual necrosis. This study sought to improve the AFG efficiency using a hydrogel as a carrier for human fat graft (F) with and without platelet-rich plasma (PRP). PRP is clinically well known for the local release of several endogenous growth factors and has been in clinical use already. A human-fat-graft-encapsulated pectin–alginate hydrogel (FG) was developed and characterized. PRP was added to F to develop a human fat graft with PRP (FP). FP was admixed with a pectin–alginate hydrogel to develop FGP. FG and FGP showed the smooth injectable, elastic, and shear-thinning properties. FG and FGP groups showed enhanced cell viability and proliferation compared to the control F in vitro. We also investigated the in vivo angiogenesis and neo-adipogenesis ability of F, FG, FGP, and FP in nude mice after subcutaneous injection. After 2 and 4 weeks, an MRI of the mice was conducted, followed by graft explantation. The explanted grafts were also assessed histologically and with immunohistochemistry (IHC) studies. MRI and histology results revealed better vascularity of the FG and FGP system compared to fat graft alone. Further, the IHC studies, CD 31, and perilipin staining also revealed better vasculature and adipogenesis of FG and FGP systems. These results indicate the enhanced angiogenesis and adipogenesis of FG and FGP. Thus, developed pectin–alginate hydrogel-based fat graft systems FG and FGP replenish the native microenvironment by mediating angiogenesis and adipogenesis, thereby maximizing the clinical outcomes of autologous fat grafting.

## 1. Introduction

Autologous fat grafting (AFG) is an inevitable revolutionizing technique employed for soft tissue augmentation following congenital, traumatic, and post-surgical loss [1,2,3,4,5,6,7,8,9]. These soft tissue deformities render patients psychologically impaired due to disfiguration of the body. AFG is a minimally invasive approach involving the injection of the patient’s own liposuctioned fat into the defect site. AFG surpasses other tools like synthetic fillers and flap reconstruction, owing to its economical, autologous nature, reduced invasiveness, and prolonged positive effects [10,11,12,13]. Currently, AFG is considered to be a standard and simple procedure to rectify varying degrees of soft tissue defects.

The regained popularity of fat grafting following liposuction has led to a search for various aspects to improve fat graft survival in adipose tissue engineering (ATE). This includes technical aspects of harvesting, processing, and preparation of sterile fat graft [14] and incorporation of hydrogels or bio factors [15,16,17] in order to increase the survivability of fat graft. The technical aspects of harvesting and preparation of fat graft through lipoaspiration have been standardized by Coleman [18,19]. Type and size of the cannula, pressure during harvesting, centrifugal speed and time, sterile collection of fat, injection site, and volume of injection are certain factors to be considered while grafting fat [20,21]. Several manual parameters governing the viability of fat graft increases the complexity during optimization. Thus, the clinical outcome of AFG exhibits higher rates of graft resorption due to poor vascularization and necrosis, resulting in multiple lipotransfers periodically [22,23].

Various strategies have been tried to improve fat graft survivability by adding of adipocyte-derived stem cells, scaffolds, hydrogels, and growth factors [16,17,24]. The state-of-the-art technology would be isolation, expansion, and seeding of adipose-derived stem cells (ADSCs) on a scaffold for adipose regeneration. Various challenges still remain in its clinical use. Apart from the laborious process involved in stem cell isolation and expansion, the safety of in vitro expanded stem cells, delivery method, possible complications of malignant transformation, and immunogenicity in allogenic transplant are some of the major challenges [25,26,27]. In adipose tissue regeneration, an injectable hydrogel appears to be a promising substrate as it mimics the extracellular matrix of various tissues, thereby supporting cell survival. Hence, the advent of an ideal hydrogel can tackle the lacunae in autologous fat transfer. The need to develop an injectable hydrogel regime with adipogenic potential for the betterment of conventional AFG has been discussed [28]. Gels including collagen, hyaluronic acid, matrigel, and fibrin have been studied to improve the survivability of fat graft [15,29,30,31,32]. Alghoul et al. used a hyaluronic acid gel with fat graft in a rat model and found markedly enhanced vascularity with early graft survival and prolonged volume maintenance [32]. Hence, efforts to look into the efficacy and safety of other injectable hydrogel systems for adipose tissue engineering in clinical use will be beneficial. Alginate-based gels aid in regulating proper infiltration of cells, nutrients, and metabolites with increased angiogenesis and adipogenesis [33,34]. Munarin et al. studied the differentiation potential of an injectable pectin-based hydrogel towards osteogenic lineage and concluded stating that pectin delayed biodegradation, which helped in stable bone formation [35]. The viscosity of the alginate and pectin can be customized to form an injectable hydrogel, which, when encapsulated with the fat graft, tends to improve the nutrient diffusion onto the graft injected and supports cell differentiation and growth. Pectin and alginate can exhibit properties similar to the natural extracellular matrix and provide excellent bioactivity when used as a scaffold to prepare hydrogel. Pectin and alginate were cross-linked with calcium chloride solution to form a hydrogel (G). Hence, in this study, we intend to use a naturally derived pectin–alginate hydrogel to encapsulate the fat graft, which can provide a conducive environment for adipose tissue regeneration. This simpler approach also negates various challenges associated with the use of expanded stem cells.

The addition of PRP, commonly employed in clinical situations along with fat graft, tends to favor the viability by reducing the resorption rate [36,37,38,39,40]. This is known to facilitate adipogenic differentiation by maintaining sustained proliferation, resulting in a constructive effect [41]. It acts as a rich reservoir of substantial growth factors, including vascular endothelial growth factor (VEGF), insulin-like growth factor-1 (IGF-1), fibroblast growth factor (FGF), epidermal growth factor (EGF), and other growth factors that are released upon activation.

**Figure 1:**
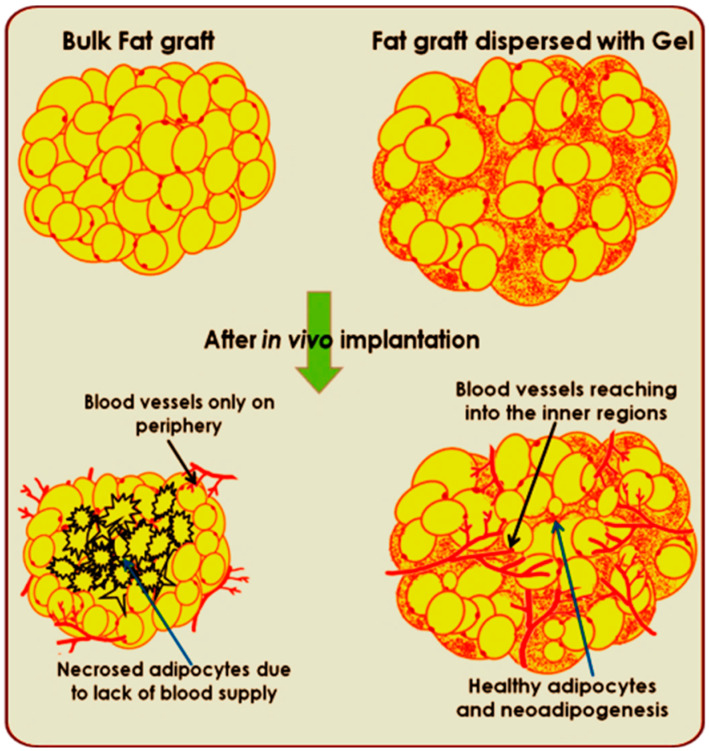
Schematic representation of pectin–alginate hydrogel incorporated human fat graft in enhancing angiogenesis and adipogenesis.

Based on the above aspects, we focused on improving the cell survival, adipogenesis and, vascularization of fat graft by encapsulating it in an injectable pectin–alginate hydrogel as shown in Figure 1. PRP added to the fat-graft-encapsulated hydrogel as a bioactive factor to supplement it with growth factors for cellular differentiation and proliferation. This study was conducted in nude mice to negate the immunological reactions as the human-fat-graft-encapsulated injectable hydrogel was used.

## 2. Materials and Methods

The fat graft and PRP were obtained from a consenting human volunteer undergoing liposuction for lipodystrophy. Institutional ethical committee approval was obtained. Alginic acid sodium salt from brown algae (4–12 cP, Low viscosity), pectin derived from citrus peel (Galacturonic acid > 74%), calcium chloride, Oil Red O, Harri’s Hematoxylin, and Eosin solution were procured from Sigma. Anti-human CD 31, anti-human perilipin, and anti-human vimentin antibodies were purchased from Abcam. α-MEM, Pen-strep, FBS, and Trypsin-EDTA were obtained from Invitrogen. Live staining kit and Quanti-iT Picogreen dsDNA assay kit were procured from Thermo Fisher Scientific.

### 2.1. Extraction of Human Fat Graft (F)

**Figure 2:**
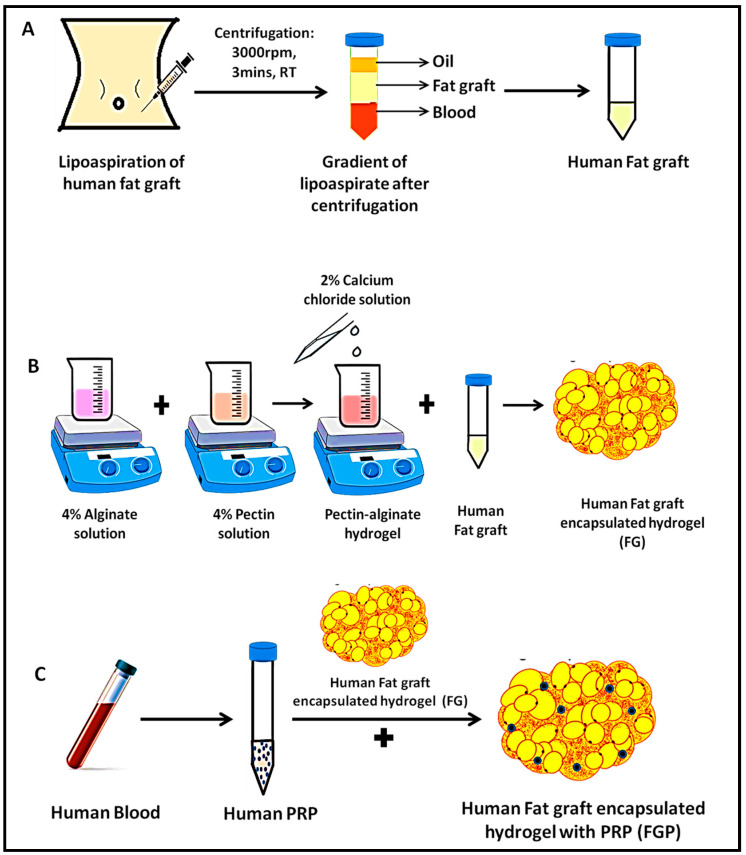
(**A**) Extraction of fat graft from human lipoaspirate; (**B**) preparation of fat-graft-encapsulated hydrogel; and (**C**) preparation of fat-graft-encapsulated hydrogel with PRP.

After infiltrating the tumescent solution, fat graft was harvested from a healthy human donor undergoing abdominal liposuction by syringe technique using a 10 mL syringe and a 3 mm blunt tip Mercedes cannula. The harvested fat graft was centrifuged at 3000 rpm for 3 min to remove the upper oily layer while the decanting lower layer of blood and tumescent solution. The middle layer containing viable adipocytes was used as the fat graft (F) as shown in Figure 2A.

### 2.2. Preparation of Fat-Graft-Encapsulated Pectin–Alginate Hydrogel (FG)

A total of 4% alginate and 4% pectin were dissolved in cell culture media (α-MEM basal media) at a volume ratio of 3:5. Both the polymeric solutions were allowed to mix thoroughly for 30 min, followed by the addition of 2 wt% calcium chloride as a cross-linking agent. The homogenous, smooth hydrogel obtained was termed pectin–alginate hydrogel (G). The fat-graft-encapsulated hydrogel (FG) was prepared by blending the fat and hydrogel at a volumetric ratio of 60:40, as illustrated in Figure 2B. The above ratio was optimized by assessing the uniform dispersibility of fat cells in the hydrogel and the injectable nature. FG was prepared under continuous stirring for homogenous distribution of components.

### 2.3. Preparation of Fat-Graft-Encapsulated Pectin–Alginate Hydrogel with PRP (FGP)

About 20 mL of whole blood was withdrawn from the same patient undergoing liposuction. Platelet-rich plasma (P) was prepared in our blood bank using a standard protocol. Fat graft with PRP (FP) was prepared at a volumetric ratio of 9:1. Fat graft with PRP-encapsulated hydrogel (FGP) was prepared by fat with PRP in the above-mentioned ratio by blending hydrogel at a volumetric ratio of 60:40, which is shown in Figure 2C.

### 2.4. Physiochemical Characterization of the Developed Hydrogel

#### 2.4.1. Inversion and Injectability

The developed hydrogel was loaded into a 1 mL syringe and injected through an 18 G needle to understand its flow behavior. The fat-graft-encapsulated hydrogel was kept in a beaker, inverted, and undisturbed to assess its solid behavior.

#### 2.4.2. Fourier-Transform Infrared Spectrometry (FTIR) Analysis

FTIR spectrometer (IR Affinity-1S, Shimadzu, Kyoto, Japan) was performed (from 500 to 4000 cm^−1^) to analyze the functional groups of alginate, pectin, and fat graft present in the hydrogel. The lyophilized fat-graft-encapsulated hydrogel (FG), fat graft (F), hydrogel (G), alginate, and pectin were pelletized with potassium bromide and analyzed using the FTIR instrument.

#### 2.4.3. Rheological Studies

The viscoelastic property of F, G, FP, FG, and FGP was evaluated using Malvern Kinexus Pro Rheometer armed with Peltier system for regulating the temperature. All the studies were performed using 20 mm parallel-plate geometry with a 0.5 mm gap at 37 °C. The injectable property of the prepared hydrogel was analyzed by flow curve in the range of 0.1 s^−1^ to 100 s^−1^. Frequency sweep analysis was conducted to determine the strength of the prepared hydrogels, which was carried out in the range of 0.1 to 10 Hz with a constant strain of 1% to measure the elastic (G’) and viscous modulus (G″). The change in elastic modulus, viscous modulus, and phase angle (δ) was compared among groups.

### 2.5. In Vitro Cytocompatibility Studies

The cytocompatibility of F, FP, FG, and FGP was performed using human umbilical vein endothelial cells (HUVECs). The cells were seeded onto the above groups with a seeding density of 10,000 cells per well of a 96-well plate. This was cultured at 37 °C in an incubator complemented with 5% CO_2_ for 24 and 48 h. After the specific time points, dsDNA was extracted from cells by incubating in lysate buffer (1% Triton X-100 in PBS) for 15 min. The dsDNA content of the cell lysate was then quantified using a fluorometric picogreen assay kit at an excitation wavelength of 490 nm and an emission wavelength of 520 nm. The DNA content of the HUVECs seeded in contact with various systems (F, FP, FG, FGP) was quantified. The dsDNA standards and samples were used in triplicate. Calcein AM staining and Oil Red O staining was carried out to assess the viability of fat cells and lipid accumulation of the developed FG system.

### 2.6. In Vivo Study

The hydrogel (G), fat graft, and its various combinations (F, FP, FG, and FGP) were injected onto the dorsum of nude mice in the subcutaneous region (1 mL) at three sites, one in the upper back and two in the lower back region using a 2 mL syringe and an 18 G needle. MRI was carried out to assess vascularity using a Bruker 7-tesla small animal magnetic resonance image coiler with Gadolinium as contrast (0.4 microliter/g of 0.5 mmol/mL of meglumine gadoterate). Nude mice were euthanized by an overdose of anesthesia (intraperitoneal phenobarbitone) at the end of the 2nd and 4th weeks. Injected fat graft groups were carefully dissected, explanted, and incubated in neutral 10% formalin solution, followed by paraffin embedding for further histological evaluation.

### 2.7. Histological Analysis

Paraffin-embedded sections of 5 μm thickness were deparaffinized and dehydrated using graded series of ethanol and were stained using hematoxylin, followed by Eosin.

### 2.8. Immunohistochemistry

Vascularity was assessed by antibodies against CD 31 as an endothelial marker, and the viability of the adipocytes was assessed by perilipin staining of injected fat graft groups [36]. Vimentin staining was conducted to confirm the species relativity as the human fat graft was injected into nude mice. Briefly, sections were dewaxed, rehydrated, and subjected to antigen retrieval using citrate buffer at pH 6.0 and blocked peroxidase enzyme using biotin, followed by primary anti-human vimentin or antibodies against CD 31 or perilipin staining. Corresponding secondary antibodies were used and counterstained using hematoxylin. Stained slides were visualized under an optical microscope (Leica Microsystems, Leica DM500, Wetzlar, Germany) that captured and quantified images.

## 3. Results

### 3.1. Preparation of Fat-Graft-Encapsulated Hydrogel

**Figure 3:**
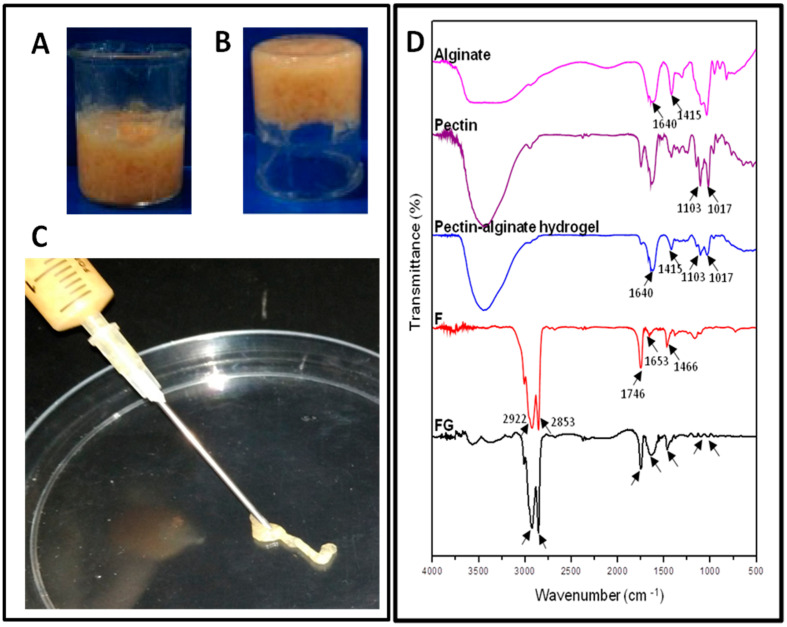
Characterization of fatgraftencapsulated hydrogel (FG); (**A**) image of FG; (**B**) inversion test of FG; (**C**) injectability of FG; and (**D**) FTIR of alginate, pectin, pectin–alginate hydrogel (G), F and FG.

The optimal concentration of the fat graft and injectable hydrogel at a ratio of 60:40 was used to form a dense porous network to encapsulate the pectin–alginate hydrogel. Divalent calcium ions act as a cross-linker forming a stable hydrogel, enhancing the smooth flow of hydrogel through the syringe of an 18G needle as depicted in Figure 3.

### 3.2. Physiochemical Characterization of the Developed Hydrogel

An inversion test was performed to confirm the gelation property of FG, and it was found to be stable (Figure 3B). The smooth flow of FG was observed through an 18G needle, confirming its injectable nature (Figure 3C). At the same time, fat graft alone was not injectable through the 18G needle. The FTIR spectrum of alginate, pectin, control G, control F, and FG is shown in Figure 3D. The characteristic absorption peaks of alginate appeared at 1640 and 1415 cm^−1^ due to asymmetric and symmetric stretching of carboxylate vibrations, respectively. Pectin showed peaks at 1017 to 1103 cm^−1^, which correspond to carboxyl groups. The pectin–alginate hydrogel (G) confirmed the presence of both pectin and alginate. The peaks of control fat (F) were observed at 2922, 1746, and 1466 cm^−1^, indicating the asymmetric stretching of –CH_2_ group, fatty acid ester, and –CH_2_ bending, respectively. The final system of the fat-graft-encapsulated pectin–alginate hydrogel (FG) speculated that all peaks corresponded to individual components of the hydrogel, confirming the encapsulation of fat graft into the hydrogel.

### 3.3. Rheological Properties of the Developed Hydrogel

**Figure 4:**
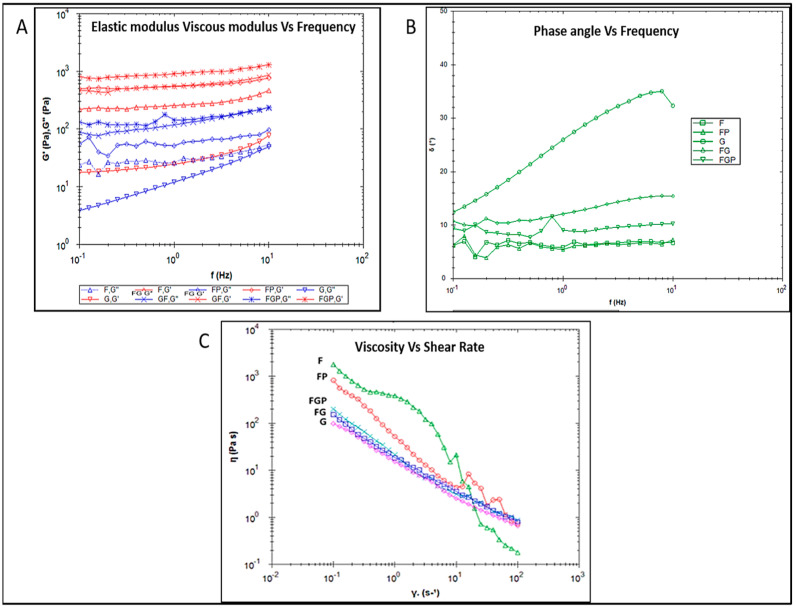
Rheological characterization of developed hydrogel; (**A**) frequency sweep analysis of elastic and viscous modulus vs. frequency; (**B**) phase angle vs. frequency; (**C**) flow curve analysis.

Viscoelastic behavior was studied to understand the injectability and strength of the prepared hydrogels. The elastic and viscous modulus was analyzed against frequency, as shown in Figure 4A. For all systems, the elastic modulus was higher than the viscous modulus, showing the elastic nature of the prepared gel system. The phase angle of all the gel systems was less than 40°, indicating the solid-like nature as shown in Figure 4B. Irregular flow curve of control fat (F) marks the sub-optimal injectable nature. Encapsulation of the fat graft into the pectin–alginate hydrogel (FG) resulted in a linear decrease in viscosity with an increase in shear rate, indicating the smooth injectable nature. The addition of PRP did not impact the injectable property of the hydrogel as shown in Figure 4C, represented by FGP. Shear-thinning behavior is the most entailed property of an injectable material and was studied by flow curve analysis, and all the gel systems exhibited the same (Figure 4C).

### 3.4. In Vitro Cytocompatibility

**Figure 5:**
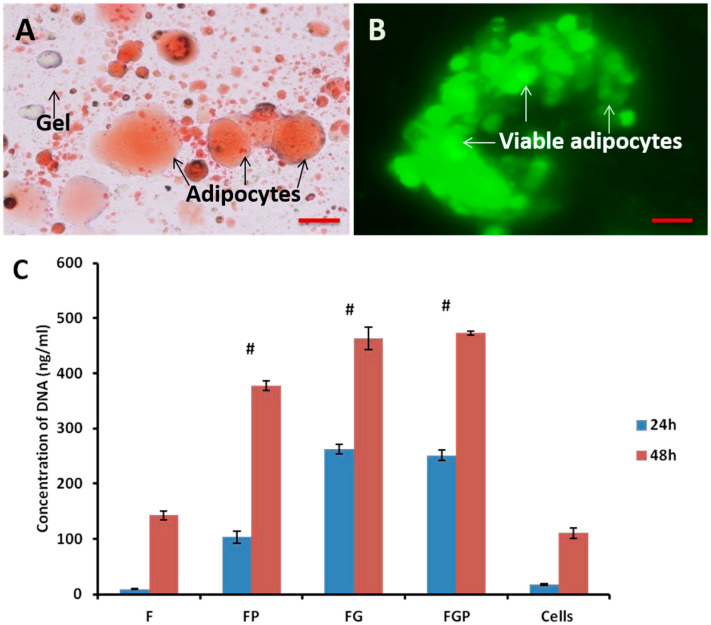
In vitro cytocompatibility of prepared hydrogel. (**A**) Oil Red O staining of FG; (**B**) live staining of adipocytes in FG; (**C**) DNA concentration (*n* = 3) of various groups (F, FG, FGP, FP); #—indicates higher cell growth at 24 and 48 h in FP, FG, and FGP groups when compared to F group (S.D. *p* < 0.05). Scale bar: 200 μm.

Accumulation of lipid droplets in FG was observed using Oil Red O staining, indicating the retained adipogenic property of fat graft after encapsulation (Figure 5A). Calcein AM-stained images of FG revealed the biocompatible nature of the hydrogel (Figure 5B). Green fluorescence stipulates that adipocytes are viable after encapsulating the hydrogel. HUVECs were used to study the proliferation of these cells cultured on the developed fat and fat–hydrogel systems (F, FP, FG, and FGP) (Figure 5C). The cell growth differences at 24 and 48 h were comparatively higher in the FP, FG, and FGP groups when compared to the F group. The FP group showed a four-fold increase in cell growth compared to FG, and the FGP group showed a two-fold increase. Even though the FP group showed enhanced cell growth, the addition of PRP to FG did not significantly impact cell growth. Thus, the pectin–alginate hydrogel system supports the cell growth of fat graft.

### 3.5. In Vivo Studies

**Figure 6:**
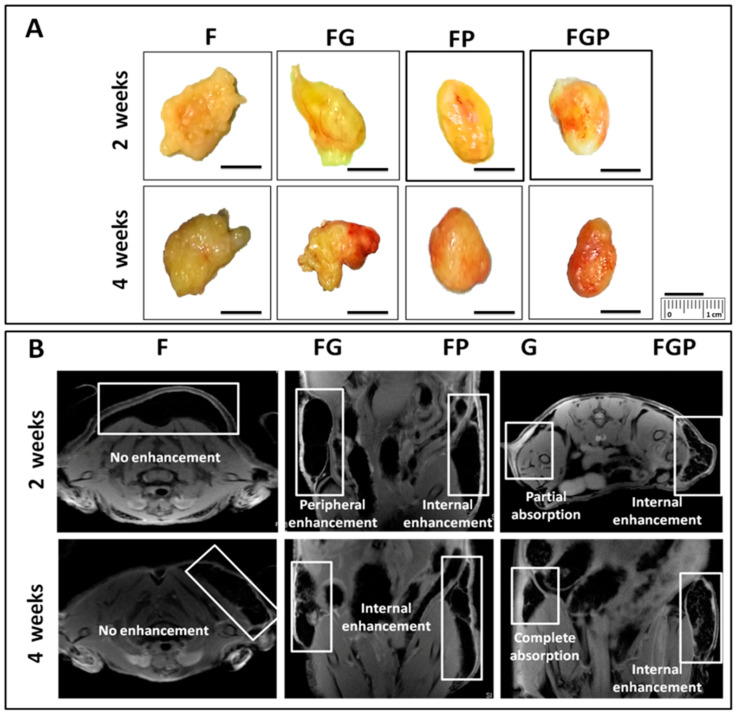
(**A**) Macroscopic appearance of subcutaneously injected explants and (**B**) MRI of F, G, FP, FG, and FGP groups at 2 and 4 weeks.

There was no inflammation, seroma, or abscess formation of grafts or adjacent tissue during this study. The specimens were harvested for histological examination (Figure 6A). The redness of explants FG, FP, and FGP grossly at 4 weeks was due to vascularity. Complete absorption of the control G group at 4 weeks was observed in MRI (Figure 6B). At 4 weeks, the FG, FGP, and FP groups showed both peripheral and internal enhancement indicative of vascularization compared to F.

### 3.6. Histological Analysis

**Figure 7:**
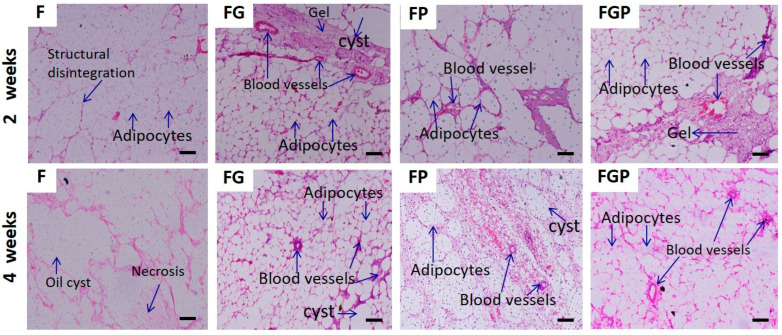
Histological examination of F, FG, FP, and FGP using H&E staining; Scale bar: 100 μm.

Histological analysis using H&E staining in Figure 7 showed blood vessel formation in the FG, FP, and FGP groups. In the F group, cysts and necrosis with cellular structural disintegrity were observed. After 2 weeks, de novo FP underwent remodeling of mature adipose tissue, whereas FG and FGP initiated neo-adipogenesis and angiogenesis. At the end of the fourth week, FG and FGP exerted well-maintained cellular architecture with adequate vascularization, indicating the hydrogel encapsulated by the fat graft had a positive effect on the viability of the fat graft.

### 3.7. Immunohistochemistry

**Figure 8:**
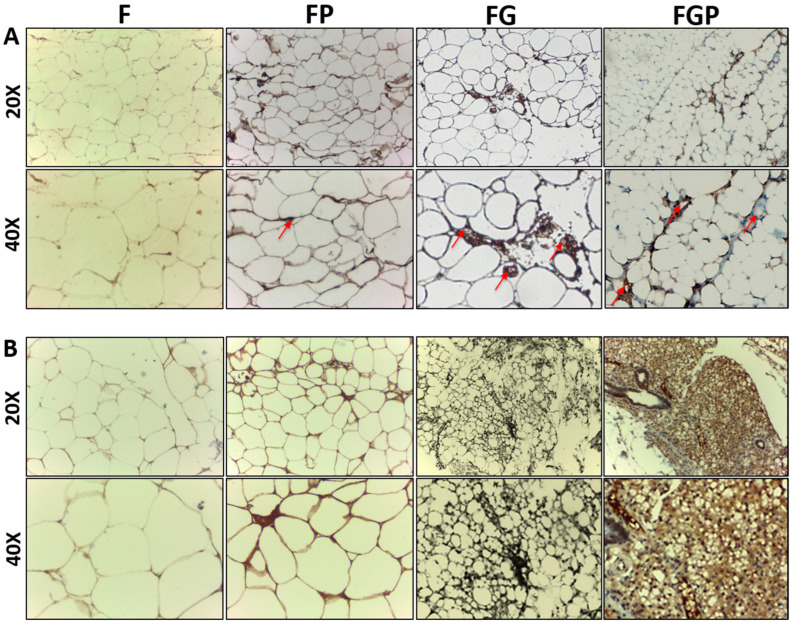
Immunohistochemical staining using (**A**) CD 31, arrow indicates the blood vessels and (**B**) perilipin at 4 weeks of F, FP, FG and FGP; G-Hydrogel; Scale bar: 100 μm (20×), 50 μm (40×).

Blood vessel formation and the endothelialization of de novo explants were analyzed using CD 31 antibody staining. FP, FG, and FGP had higher ingrowth of numerous capillaries compared with the F group at 4 weeks (Figure 8A). The F group appeared to have disintegrated adipocyte morphology with less vascularity. Perilipin stained the accumulation of lipid droplets surrounding adipocytes, as mentioned in (Figure 8B). The staining intensity was higher in FG and FGP, indicating increased adipocyte differentiation in both the second and fourth weeks. The fat grafts (F) had necrosis, evidenced by structural disintegrity and weak perilipin staining. The improved fat graft survival was exhibited in FG, and FGP proved the collaborative effect of angiogenesis and adipogenesis. Thus, the key role of hydrogel in providing mechanical support for the improvisation of cell survival has been shown to have a positive impact.

**Figure 9:**
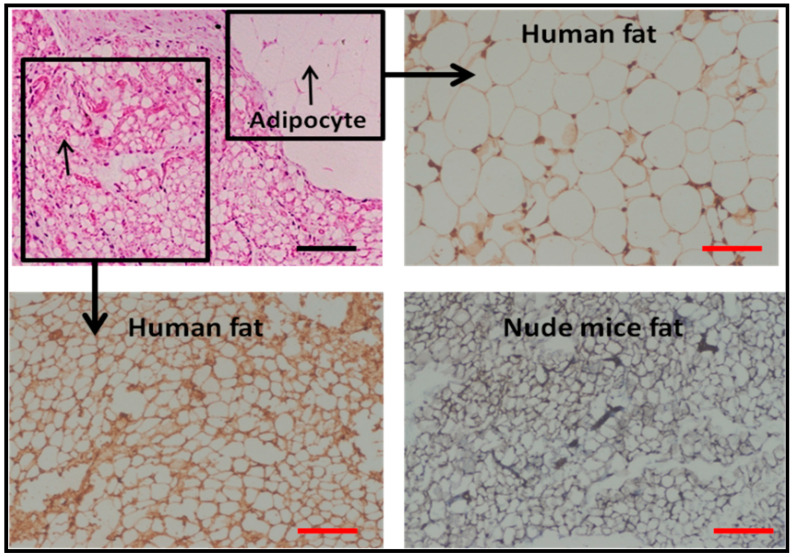
Vimentin staining of nude mice fat graft and FG; Scale bar: 100 μm.

To confirm the origin of the newly formed adipocytes, vimentin staining was carried out. The human adipocytes stained positive, whereas nude mice adipocyte did not, thus ensuring the neo-adipogenesis of the human fat graft (Figure 9).

## 4. Discussion

The in vivo study of the developed fat-graft-encapsulated pectin–alginate hydrogel showed improved outcomes in fat grafting. This dynamic system can improve the current autologous fat grafting (AFG) scenario by minimizing undesirable effects, such as necrosis, and resorption. Though several studies focused on increasing cell survival, there exists a void on a simpler and mechanistic approach towards AFG.

Low methoxy pectin and alginate undergo sol–gel phase transition via classical scalar percolation theory, and their synergistic effect tends to mold the cellular behavior within hydrogels [42,43,44]. The pectin–alginate system acts as a good carrier and reservoir of bioactive supplements and factors, thereby governing the proper infiltration and migration of cells and biomolecules. Calcium chloride acts as a vital cross-linker between the copolymeric hydrogel and fat graft, as well as a proficient activator of PRP. Braccini et al. has demonstrated an “egg-box model” of calcium-induced gelation of pectin and alginate by forming junction zones by inducing dimerization and aggregation [45]. Cavallo et al. evaluated the potential of calcium chloride to activate platelets and observed sustained activation for a period of 24 h [46]. They also used thrombin as a cofactor with calcium chloride to minimize blood clot formation. Immediate gelation was observed due to the presence of divalent calcium ions.

The injectable nature of the developed system is of utmost importance in this study as the intention is to deliver in a minimally invasive fashion. The shear-thinning ability of a hydrogel determines the injectable nature of the material for filling defective sites. It was found that all the hydrogel groups retained their strength with minimal variations over a fixed frequency range, even under constant strain (Figure 4C). Fat graft group F did not possess the desirable injectable property, leading to clogging of fat depots with discontinuous flow and a sudden release of excess volume than required. The hydrogel-incorporated fat graft showed better injectability (Figure 3C), an added advantage clinically.

In vivo vascularity and adipogenic ability followed the same trend as in vitro cell proliferation. The origin of neo-adipogenesis when injecting the human fat graft into the immune-deficient nude mice was questionable as to whether it is of human or mice origin. The neo-adipogenesis could be of either pre-existing preadipocytes of human fat graft or due to recruitment of endogenous precursors from the host, i.e., nude mice. Contrary to the study conducted by Stillaert et al., vimentin staining, as shown in Figure 9 of our study, showed the origin of neo-adipogenesis is of human origin rather than host origin [47].

Haug et al. studied the effect of HUVECs on neovascularization in an adipogenic mouse model for 6 months. They found that HUVECs play a significant role in stabilizing vessel formation [48]. PRP-containing growth factors aid in endothelial cell proliferation, thereby amplifying angiogenesis and adipogenesis. Hausman et al. studied the fundamental aspects of adipogenesis and angiogenesis and concluded by stating that adipogenesis may be regulated by similar factors driving angiogenesis [49]. The FG, FP, and FGP groups expressed enhanced adipogenesis and vascularization compared to the F group (Figure 8). This may be due to poor diffusion of nutrients and necrosis to the fat graft in the F group. The better adipogenesis in the additive groups of the hydrogel and/or PRP owe to the supportive nature, improved nutrient diffusion with the hydrogel, and the positive effect of growth factors in the PRP. Fukumura et al. studied the paracrine regulation of angiogenesis and adipogenesis. Surprisingly, inhibition of VEGF reduced both angiogenesis and adipogenesis by blocking preadipocyte differentiation. It was also found that paracrine interaction between endothelial cells and preadipocytes leads to a reciprocative effect on both angiogenesis and adipogenesis [50].

The developed FG hydrogel is speculated to provide a conducive microenvironment that encourages cell migration, proliferation, and differentiation. Adrian et al. demonstrated the effect of angiogenic growth factors with the commercially available extracellular matrix during adipogenesis. The combination of growth factors significantly fortified the adipogenic ability of ADSCs [51,52]. The addition of PRP to FG does not significantly impact adipogenesis and angiogenesis, as shown in in vitro (Figure 5C) and in vivo studies (Figure 7 and Figure 8). This negates the addition of PRP in this system, as th e pectin–alginate hydrogel system exerts more beneficial effect than PRP. This may be because the developed hydrogel helps in tuning the hydrophilicity, mechanical, and mass transport properties of the encapsulated fat graft. Overall, the developed hydrogel provides the mechanical framework to support cell growth, whereas PRP provides various growth factors for the upregulation of angiogenesis and adipogenesis, thereby minimizing the debilitating nature of the conventional method of AFG.

Autologous fat graft being a rich source of ADSCs, preadipocytes, and adipocytes, with the hydrogel, serves as the next-generation candidate for carrying bioactive factors necessary for adipogenesis. In the conventional method of AFG, the fat graft survives by nutrient and oxygen diffusion during the initial 48–72 h until neovascularization occurs but is restricted to peripheral sites, resulting in necrosis of the inner core of the fat graft [53]. As neovascularization initiates, the survival of preadipocytes and ADSC increases, resulting in adequate neo-adipogenesis. The mature adipocytes in the avascular fat graft can lead to necrosis and volumetric reduction in a naturally occurring degenerative remodeling process within the human body. When used alone, the fat graft tends to enter an ischemic phase where mature adipocytes are susceptible to lipophage due to a lack of oxygen diffusion and nutrients. The possible mechanism for enhanced angiogenesis and neo-adipogenesis in the fat-graft-encapsulated hydrogel is possibly due to improved nutrient diffusion and endothelial progenitors’ migration.

The pectin–alginate hydrogel system provides the hydrophilic environment, facilitating the diffusion of the nutrients and thereby extending adipocyte viability. The hydrophilic nature of the hydrogel has the potential to absorb nutrients from the surrounding tissues and to diffuse gases and metabolites, enriching the core of the fat graft. The admixing of the hydrogel with fat graft encapsulates the cells, protecting them from inflammatory cells [32]. It also creates an intercellular space, preventing the clumpiness of adipocytes, thereby minimizing fat necrosis. Due to its extracellular-matrix-mimicking property, it also maintains the morphology and function of mature adipocytes. Thus, the hydrogel creates a better niche for ADSCs and endothelial progenitor cell migration, proliferation, and differentiation, resulting in reciprocal regulation of neovascularization and neo-adipogenesis.

## 5. Conclusions

In this study, a human fat graft encapsulated into a pectin–alginate hydrogel was developed to enhance the adipogenesis and angiogenesis of fat graft. The prepared hydrogel is stable, injectable, and has a shear-thinning property. Biocompatibility of the prepared F-containing hydrogel system showed enhanced cell growth. In vivo MRI imaging showed enhanced vascularity of the fat-graft-containing gel system compared to F alone. Histology and immunohistochemistry also revealed better angiogenesis and adipogenesis of the fat-graft-encapsulated hydrogel system. Vimentin staining proved that neo-adipogenesis is of human origin. Hence, this dynamic hydrogel system can act as a carrier for fat graft in AFG to overcome the current drawback of resorption. This fat-graft-encapsulated pectin–alginate hydrogel system has potential applications in soft tissue regeneration.

## Data Availability

No new data were created or analyzed in this study. Data sharing is not applicable to this study.

## Abbreviations

AFG	Autologous fat grafting
G	Pectin–alginate hydrogel
F	Human fat graft
PRP	Platelet-rich plasma
FG	Human-fat-graft-encapsulated pectin–alginate hydrogel
FGP	Human-fat-graft-encapsulated pectin–alginate hydrogel
IHC	Immunohistochemistry studies
ATE	Adipose tissue engineering
VEGF	Vascular endothelial growth factor
IGF-1	Insulin-like growth factor-1
FGF	Fibroblast growth factor
EGF	Epidermal growth factor
FTIR	Fourier-transform infrared spectrometry
HUVECs	Human umbilical vein endothelial cells
ADSCs	Adipose-derived stem cells
